# Activation of Distinct Channelrhodopsin Variants Engages Different Patterns of Network Activity

**DOI:** 10.1523/ENEURO.0222-18.2019

**Published:** 2020-01-02

**Authors:** Na Young Jun, Jessica A. Cardin

**Affiliations:** 1 Department of Ophthalmology, Yale University, New Haven, CT 06520; 2 Department of Neuroscience, Yale University, New Haven, CT 06520; 3 Kavli Institute for Neuroscience, Yale University, New Haven, CT 06520

**Keywords:** Channelrhodopsin, Chrimson, Chronos, cortex, γ oscillations, optogenetics

## Abstract

Several recently developed Channelrhodopsin (ChR) variants are characterized by rapid kinetics and reduced desensitization in comparison to the widely used ChR2. However, little is known about how varying opsin properties may regulate their interaction with local network dynamics. We compared evoked cortical activity in mice expressing three ChR variants with distinct temporal profiles under the CamKII promoter: Chronos, Chrimson, and ChR2. We assessed overall neural activation by measuring the amplitude and temporal progression of evoked spiking. Using γ-range (30–80 Hz) local field potential (LFP) power as an assay for local network engagement, we examined the recruitment of cortical network activity by each tool. All variants caused light-evoked increases in firing *in vivo*, but each demonstrated different temporal patterning of evoked activity. In addition, the three ChRs had distinct effects on cortical γ-band activity. Our findings suggest the properties of optogenetic tools can substantially affect their efficacy *in vivo*, as well their engagement of circuit resonance.

## Significance Statement

Genetically modified opsins are some of the most widely used experimental tools in modern neuroscience. However, although these tools are well characterized at the single-cell level, little is known about how the varying properties of the opsins affect their interactions with active neural networks *in vivo*. Here, we present data from experiments using three optogenetic tools with distinct activation/inactivation and kinetic profiles. We find that opsin properties regulate the amplitude and temporal pattern of activity evoked *in vivo*. Despite all evoking elevated spiking, the three opsins also differentially regulate cortical γ oscillations. These data suggest that the kinetic properties of optogenetic tools interact with active neural circuits on several time scales. Optogenetic tool selection should therefore be a key element of experimental design.

## Introduction

The advent of easily accessible optogenetic tools for manipulating neural activity has substantially altered experimental neuroscience. The current optogenetics toolkit for neuroscience comprises a large number of Channelrhodopsins (ChRs), Halorhodopsins, and Archaerhodopsins that enable activation and suppression of neural activity with millisecond-timescale precision. Within the ChR family, many variants have now been made with altered activation spectra, photocycle kinetics, and ion selectivity. The first tool to be widely used in neuroscientific approaches, ChR2, is a nonspecific cation channel with sensitivity to blue light. ChR2 conferred the ability to evoke action potentials with high precision and reliability across a wide range of cell types (Boyden et al., 2005; Cardin et al., 2009; Deisseroth, 2015). However, the utility of this tool has been somewhat limited by its relatively long offset kinetics and fairly rapid inactivation of photocurrents in response to sustained strong light stimulation (Boyden et al., 2005; Bamann et al., 2008; Ritter et al., 2008; Schoenenberger et al., 2011). In addition, most naturally occurring ChRs are sensitive to blue-green light, presenting a challenge to the use of multiple tools for simultaneous optogenetic control of distinct neural populations. A significant effort in the field has therefore been made to develop ChR variants with faster on- and offset temporal kinetics, less desensitization over time, and red-shifted activation spectra.

Previous work has suggested that the ChRs are highly effective tools for probing the cellular interactions underlying intrinsically generated patterns of brain activity. Stimulation of parvalbumin (PV)-expressing interneurons in the cortex via ChR2 evokes γ oscillations, entrains the firing of excitatory pyramidal neurons, and regulates sensory responses (Cardin et al., 2009; Sohal et al., 2009). Similarly, ChR2 stimulation of PV^+^ GABAergic long-range projection neurons in the basal forebrain generates γ-range oscillations in frontal cortex circuits (Brown and McKenna, 2015). Recent work further suggests that ChR2 activation of Somatostatin-expressing interneurons, which synapse on both PV^+^ cells and excitatory neurons, evokes cortical oscillations in a low γ range (Veit et al., 2017). Sustained depolarization of excitatory sensory cortical neurons via ChR2 activation likewise evokes γ oscillations, likely by engaging reciprocal interactions with local GABAergic interneurons. (Adesnik and Scanziani, 2010). In comparison, activation of pyramidal neurons in mouse motor cortex via ChRGR, another ChR variant, evokes activity in a broad range of lower-band frequencies (Wen et al., 2010). High-fidelity spiking recruited by Chronos, oChiEF, and ReaChR has been used *in vitro* and *in vivo* in visual cortex (Chaigneau et al., 2016; Hass and Glickfeld, 2016; Ronzitti et al., 2017) and the auditory midbrain (Guo et al., 2015; Hight et al., 2015), but the impact of such stimulation on the surrounding network remains unclear.

Despite the substantial increase in available ChR variants with diverse kinetic and spectral properties, it remains unclear how these properties interact with endogenous temporal patterns of neural circuit activity like γ oscillations *in vivo*. Furthermore, the properties of optogenetic tools are typically validated using short pulses of light (1–100 ms) under quiet network conditions *in vitro*, but these tools are widely used for sustained neural activation (100's of ms to s) under active network conditions *in vivo* (Adesnik and Scanziani, 2010; Bortone et al., 2014; Phillips and Hasenstaub, 2016; Burgos-Robles et al., 2017). Here, we tested the impact of optogenetic tool properties on evoked activity patterns in the intact brain. We took advantage of the well-characterized γ oscillation rhythm in mouse primary visual cortex *in vivo* (Adesnik and Scanziani, 2010; Niell and Stryker, 2010; Vinck et al., 2015) as a metric for optogenetic recruitment of local network activity. Using optogenetic activation of excitatory pyramidal cells as a paradigm to evoke both spiking and cortical γ oscillations, we compared three ChRs with robust photocurrents but distinct kinetic profiles: Chronos, with high-speed on and off kinetics (Klapoetke et al., 2014); ChR2, with fast on but relatively slow off kinetics (Boyden et al., 2005); and Chrimson (Klapoetke et al., 2014), with slow on and off kinetics. We found that these tools, although expressed in the same cell types in the same brain region and effective at eliciting action potentials, evoked distinct patterns of activity and had different effects on γ activity. Together, our data suggest that the kinetic properties of engineered opsin tools affect optogenetic interactions with local circuit activity and should be a key factor in experimental design.

## Materials and Methods

### Animals

All animal procedures were performed in accordance with the Yale University Institutional Animal Care and Use Committee animal care committee’s regulations. We used both female and male C57BL/6J mice ranging from three to five months old.

### Surgical procedures

To express ChR2, Chronos, and Chrimson in pyramidal neurons, we injected AAV5-CAMKII-ChR2-GFP (Addgene # 26969), AAV5-CAMKII-CHRONOS-GFP (Addgene # 58805), or AAV5-CAMKII-CHRIMSON-GFP (Addgene # 62718), respectively, in the cortex of C57BL/6J mice. For the virus injection surgery, 1 μl of AAV was injected through a small burr hole craniotomy in the skull over the left visual cortex (–3.2 mm posterior, –2.5 mm lateral, –500 μm deep relative to bregma) using a glass pipette. Injections were made via beveled glass micropipette at a rate of ∼100 nl/min. After injection, pipettes were left in the brain for ∼5 min to prevent backflow. Mice were given four weeks for virus expression before experiments.

### Electrophysiological recordings

Mice were anesthetized with 0.3–0.5% isoflurane in oxygen and head-fixed by cementing a titanium headpost to the skull with Metabond (Butler Schein). All scalp incisions were infused with lidocaine. A craniotomy was made over primary visual cortex and electrodes were lowered through the dura into the cortex. All extracellular multiunit (MU) and local field potential (LFP) recordings were made with an array of independently moveable tetrodes mounted in an Eckhorn Microdrive (Thomas Recording). Signals were digitized and recorded by a Digital Lynx system (Neuralynx). All data were sampled at 40 kHz. All LFP recordings were referenced to the surface of the cortex (Buzsáki et al., 2012; Herreras, 2016). LFP data were recorded with open filters and MU data were recorded with filters set at 600–9000 Hz.

Optogenetic stimulation was provided via an optical fiber (200 μm) coupled to a laser (Optoengine) at either 470 nm (ChR2 and Chronos stimulation) or 593 nm (Chrimson stimulation). In each experiment, the fiber was placed on the surface of the dura over the virus injection site and the tetrodes were placed immediately posterior to the fiber.

During each experiment, a total of 150 laser pulses (470 or 593 nm) of 1.5-s duration were given at varying light intensities (0.5–10 mW/mm^2^) with 10-s interpulse intervals to allow detection of both transient and sustained spiking and LFP activity in response to light pulses. Bouts of 30 pulses were separated by 5-min baseline periods.

### Histology

Mice were perfused with 0.1 M PBS followed by 4% PFA in 0.1 M PBS. After perfusion, brains were postfixed for 8 h in 4% PFA. Brains were sliced at 40 μm on a vibratome (Leica) and mounted on slides with DAPI mounting solution (Vector). Initial images were taken with a 10× objective on an Olympus microscope and the channels were merged using ImageJ (NIH). Laminar distribution of opsin expression was estimated based on DAPI staining. Confocal images for cell counts were taken with a 64× oil objective on a Zeiss LSM 800 confocal microscope. Tissue was stained for NeuN (1:500; MAB377; Millipore) using a red secondary antibody (1:1000; Alexa Fluor Plus 594 goat anti-mouse; Invitrogen). For each mouse, NeuN^+^ cells that were positive and negative for the GFP-tagged opsin were counted in three fields of view in each layer (layers 2/3 and 5).

### Data analysis

Data were analyzed using custom scripts written in MATLAB (The MathWorks) and Python. Spikes were detected from the MU recordings using a threshold of +3 SD above the mean, where both the mean and SD were calculated from 10 s of recording preceding any light stimulation. Detected spikes were then used to calculate peristimulus time histogram (PSTH) and raster plots for visualization of optically evoked spiking. For each tetrode recording site, a two-tailed paired *t* test was performed on the firing rates in the prestimulus baseline and during light pulses to determine the presence or absence of a light-evoked change in firing rate. Sites with significant evoked firing rate changes were selected for further analysis. For each recording site, firing rates were normalized to spontaneous firing in the initial prestimulation period and the latency to peak evoked firing after light pulse onset was obtained by selecting the 10-ms interval with the highest spike counts.

Interspike intervals (ISI) were calculated as the time interval between successive spikes, and cumulative distributions of ISIs during the light pulses and spontaneous activity before the light pulses were calculated for each data set. Paired ratio measurements were taken for each light intensity. For measurements of the effect of optogenetic stimulation on mean firing rate, we measured the ratio between the prestimulus baseline (1-s period before each light pulse) and evoked firing (1-s period during each light pulse).

Spectrograms of LFP activity were obtained using 400-ms-long Hann windows sliding by 10 ms. Before short-time Fourier transform (STFT), the mean was subtracted to remove DC bias. Each trial was normalized by dividing by the RMS amplitude of the 1-s window preceding onset of the light pulse. Spectrograms were averaged across the LFP responses to 30 pulses of 10 mW/mm^2^ of 1.5-s duration. Relative power in the frequency band of interest was then calculated per frequency bin, setting the average power in the first 0.5 s in each bin to be 1. To determine whether optogenetic stimulation changed power in the γ range (30–80 Hz), we compared the average γ power during stimulation at 10 mW/mm^2^ with the average γ power during spontaneous activity before the light pulses. To further evaluate the changes in γ-range activity evoked by optogenetic stimulation, we calculated the ratio of the power spectral density in this frequency band during baseline and all stimulation conditions.

The LFP signals included low-amplitude, additive line noise at 60 Hz. The method used by Burns et al. (2010) was not applicable because (1) the amplitude difference caused by the line noise at 60 Hz in the full spectrum was not significant enough, and (2) the 60-Hz line noise was wide-band and leaked to the neighboring frequency bins of the spectrogram as well. Instead, we noticed that the line noise caused a constant phase shift at the 60-Hz line on the spectrogram. The amplitude and phase of the line noise was estimated from the mean value of the complex spectrogram at 60 Hz over all time bins during the 4-s interval, assuming that the true 60-Hz signal coming from LFP would not have a significant phase bias over the period. When we subtracted the estimated line noise from the 60-Hz frequency bin as well as the two neighboring bins, this method effectively eliminated the artifact on the spectrogram coming from the 60-Hz line noise.

### Statistics

All analyses were performed on an animal-wise basis throughout, and all data are denoted as mean ± SEM. For some comparisons, a one-way ANOVA or Kruskal–Wallis test was used, followed by a Dunn’s *post hoc* test where appropriate. In cases where nonparametric statistics were appropriate due to non-normal data distributions, a two-tailed Wilcoxon signed-rank test or the Kruskal–Wallis test were used.

## Results

### Cell type-specific expression of ChRs in mouse visual cortex

**Figure 1.**
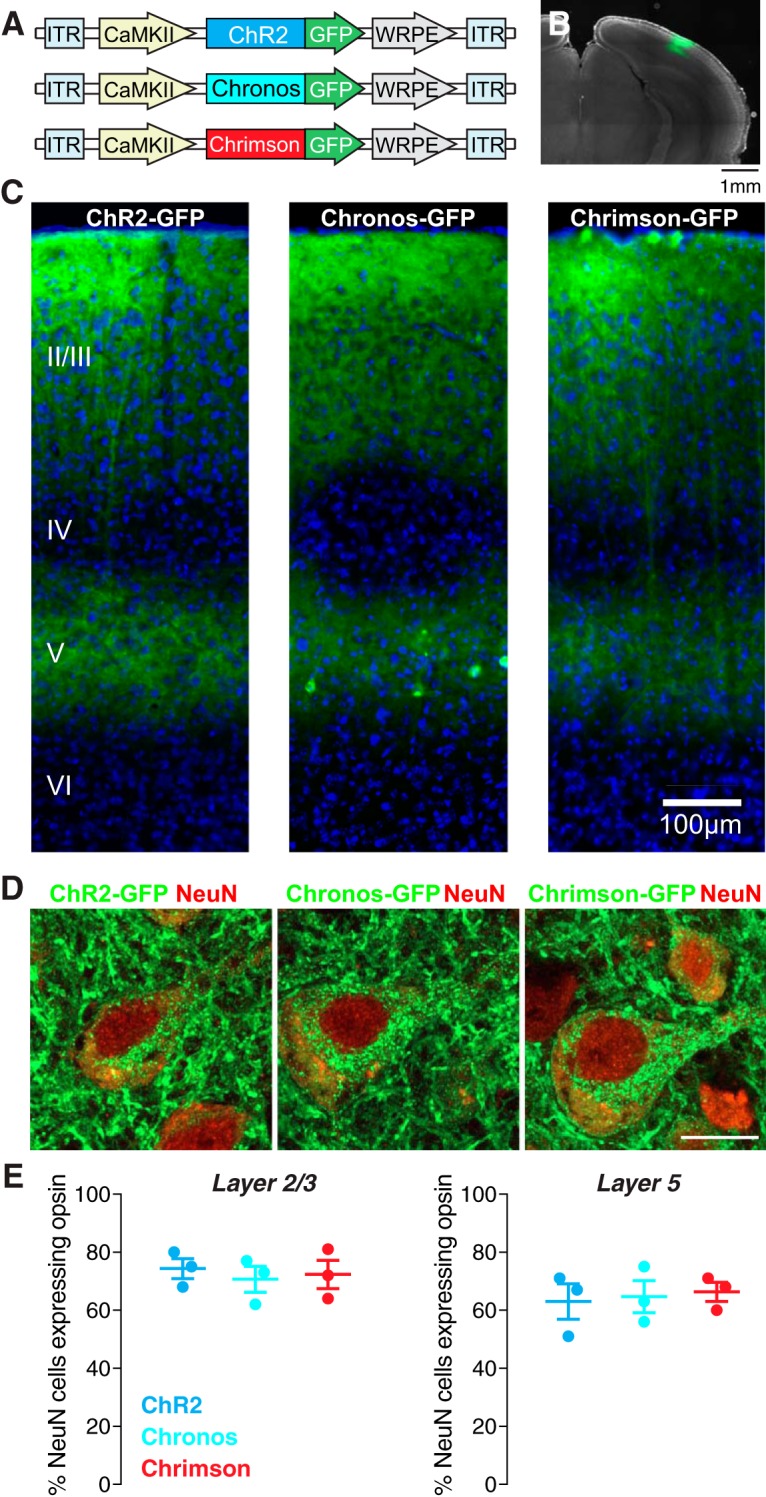
Expression of three ChR variants in excitatory neurons of the mouse visual cortex. ***A***, AAVs carrying three opsins were injected into primary visual cortex of wild-type mice; ITR, inverted terminal repeat; WRPE, woodchunk hepatitis B virus post-transcriptional element. ***B***, Spread of viral infection in the cortex. Example image showing GFP expression (green) around the area of a cortical injection of AAV5 carrying the Chronos construct. Magnification: 4×. ***C***, ChR2-GFP, Chronos-GFP, and Chrimson-GFP were robustly expressed in excitatory neurons in cortical layers 2, 3, and 5, as confirmed by DAPI staining (blue). Magnification: 10×. ***D***, Example confocal images showing expression of the three ChR variants (green) in layer 5 pyramidal neurons stained for the neuronal marker NeuN (red). Scale bar: 10 μm. Magnification: 64×. ***E***, Quantification of expression in layers 2/3 (left) and 5 (right) for each opsin.

To understand the efficacy and utility of recently developed ChR variants with differing kinetic properties, we compared three tools: ChR2, Chronos, and Chrimson (Fig. 1*A*). We expressed each tool using an AAV construct, under the control of the excitatory neuron-specific CaMKII promoter, into the visual cortex of wild-type mice. Four weeks after virus injection, each of the three ChRs was robustly expressed in a characteristic distribution of excitatory pyramidal neurons in cortical layers 2/3, 5, and 6 (Fig. 1*C*; Longson et al., 1997; Cardin et al., 2009). In each case, opsin expression was widespread in visual cortex, covering a distance of up to 410 μm from the initial injection site (Fig. 1*B*). To quantify the expression of each opsin, we performed confocal microscopy of tissue co-stained for the neuronal marker NeuN (Fig. 1*D*). We observed similar numbers of opsin-expressing neurons in layers 2/3 and 5 across all three groups of animals (layer 2/3: ChR2 74.3 ± 3.5%, Chronos 70.7 ± 4.5%, Chrimson 72.3 ± 4.9%; Kruskal–Wallis test *p* = 0.87, dF = 2, *H* = 0.36; layer 5: ChR2 63 ± 6.1%, Chronos 64.7 ± 5.5%, Chrimson 66.3 ± 3.3%; *p* = 0.92, dF = 2, *H* = 0.2; Fig. 1*E*).

### Different ChRs evoke distinct cortical activity profiles *in vivo*

The temporal profile of circuit activity evoked by different opsins may differentially engage network dynamics. To assess the initial and sustained levels of spiking evoked by each opsin, we recorded population MU and LFP activity at multiple cortical sites around each viral injection (ChR2: 18 sites in six animals, Chronos: 11 sites in five animals, Chrimson: 15 sites in six animals). Spontaneous firing rates did not differ among the three groups of animals (ChR2: 37.7 ± 5.4, Chronos: 72.8 ± 21.32, Chrimson: 42.9 ± 7.4; one-way AVOVA, *p* = 0.14, dFn = 2, dFd = 14, *F* = 2.28).

**Figure 2.**
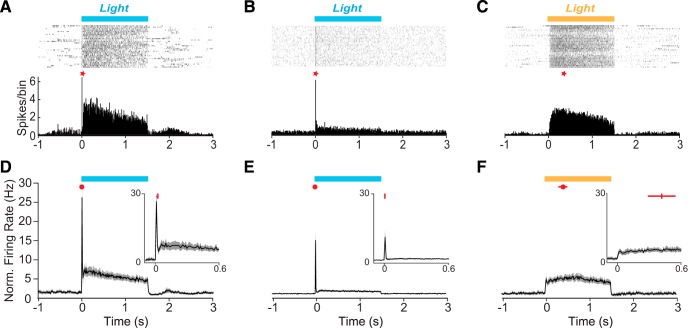
Different ChRs evoke cortical activity with distinct temporal profiles *in vivo*. ***A***, Raster plots (upper) and histograms (lower) of example MU spike activity during stimulation of excitatory pyramidal neurons with ChR2. The 1.5-s-long interval of light stimulation (10 mW/mm^2^) is indicated as shaded box. An asterisk indicates the peak firing evoked by the light pulse. ***B***, Same as in ***A***, for Chronos. ***C***, Same as in ***A***, for Chrimson. ***D***, Average PSTH for all recorded sites in ChR2-expressing mice. Red symbols and lines indicate the mean peak time and SEM of the peak time, respectively. Inset shows the initial period if evoked firing in the first 600 ms of light stimulation. ***E***, Same as in ***D***, for Chronos. ***F***, Same as in ***D***, for Chrimson.

When stimulated with 1.5 s of continuous light in an appropriate wavelength (10 mW/mm^2^, wavelength: 470 nm for ChR2 and Chronos, 593 nm for Chrimson), all three ChRs evoked sustained firing (Fig. 2*A–C*). However, each opsin was associated with a distinct temporal profile of spiking. Whereas stimulation of ChR2-expressing (Fig. 2*A*,*D*) or Chrimson-expressing (Fig. 2*C*,*F*) neurons evoked sustained firing over ∼1–2 s, stimulation of Chronos-expressing neurons generated strong initial spiking followed by a decrease toward baseline firing levels (Fig. 2*B*,*E*). The peak firing evoked by ChR2 and Chronos was rapid and reliable, whereas the peak firing achieved by Chrimson stimulation was delayed and highly variable (Fig. 2*D–F*, inset panels).

To quantify these differences in temporal kinetics induced by the three ChR variants, we compared the time between the light pulse onset and the center of the 10 ms interval with the most frequent spikes, averaged over all recording sites and mice for each ChR variant. Chronos showed the shortest peak latency of 0.005 ± 0.001 s, whereas Chrimson had a peak latency of 0.42 ± 0.13 s, compared to ChR2 at 0.01 ± 0.01 s. (Fig. 2*D–F*; Kruskal–Wallis test; *p* = 0.0015, dF = 2, *H* = 10.74). The latency to peak was longer for Chrimson, but not Chronos, compared to ChR2 (Dunn’s *post hoc* test; *p* = 0.04, *p* = 0.75).

**Figure 3.**
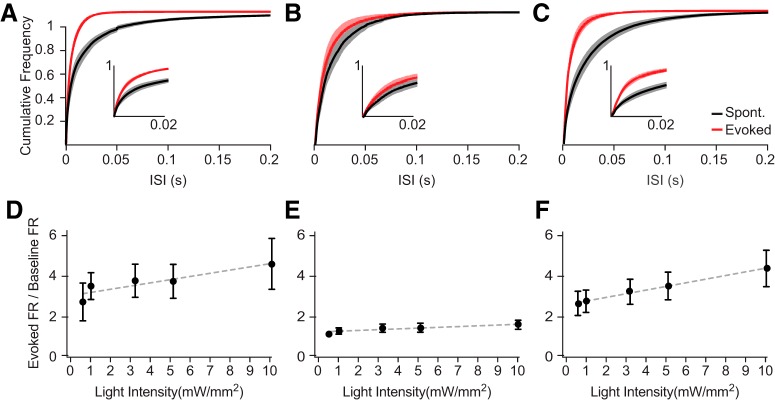
Amplitude and frequency distribution of evoked spike response varies with optogenetic tool. ***A***, ISIs of spontaneous (black) and evoked (red) MU activity during optogenetic stimulation (10 mW/mm^2^) in ChR2-expressing cortex. Inset shows an enlarged plot of the initial 200 ms of the evoked spike response. ***B***, Same as in ***A***, for Chronos. ***C***, Same as in ***A***, for Chrimson. Error bars denote SEM. ***D***, Firing rates evoked by ChR2 stimulation over a range of intensities, divided by baseline spontaneous firing immediately before the light pulses. ***E***, Same as in ***D***, for Chronos. ***F***, Same as in ***D***, for Chrimson. Dashed lines indicate linear regression of the data. Error bars denote SEM.

**Figure 4.**
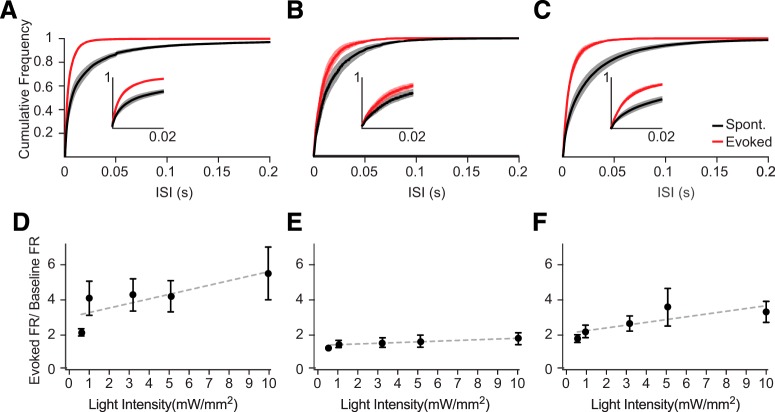
Distinct changes in evoked spike patterns during the first 100 ms after light onset. ***A***, ISIs of spontaneous (black) and evoked (red) MU activity during the initial 100 ms of optogenetic stimulation (10 mW/mm^2^) in ChR2-expressing cortex. ***B***, Same as in ***A***, for Chronos. ***C***, Same as in ***A***, for Chrimson. Error bars denote SEM. ***D***, Firing rates evoked by the initial 100 ms of ChR2 stimulation over a range of intensities, divided by baseline spontaneous firing rates immediately before the light pulses. ***E***, Same as in ***D***, for Chronos. ***F***, Same as in ***D***, for Chrimson. Dashed lines indicate linear regression of the data. Error bars denote SEM.

To assay the efficacy of each optogenetic tool in engaging local cortical neurons, we compared the recruitment of spikes in response to a range of low illumination intensities. We examined the difference in spike timing between spontaneous firing and optogenetic stimulation periods by comparing the distributions of ISIs evoked by activation of ChR2, Chronos, and Chrimson (Fig. 3). ChR2 (Fig. 3*A*) and Chrimson (Fig. 3*C*) both evoked a robust decrease in ISI, consistent with the sustained increase in firing rate, whereas activation of Chronos (Fig. 3*B*) had only a modest effect on the overall ISI distribution. ChR2-expressing (Fig. 3*D*) and Chrimson-expressing (Fig. 3*F*) mice showed increasing evoked spiking as the light stimulation intensity increased (linear regression slopes: ChR2 = 0.16 ± 0.04, *r* ^2^ = 0.82, *p* = 0.04, dFn = 1, dFd = 3, *F* = 13.56; Chrimson = 0.18 ± 0.01, *r* ^2^ = 0.9, *p* < 0.0001, dFn = 1, dFd = 3, *F* = 969.1). However, mean firing rates evoked by Chronos activation did not increase with light intensity (linear regression slope: 0.04 ± 0.01, *r* ^2^ = 0.99, *p* = 0.01, dFn = 1, dFd = 3, *F* = 28.1). Restricting analysis to the first 100 ms of the stimulation period revealed the decreased ISIs initially evoked by all three opsins (Fig. 4*A–C*). However, the profiles of evoked firing responses across increasing light intensities were similar to those observed in the full analysis shown in Figure 3*D–F* (linear regression slope: ChR2 = 0.26 ± 0.1, *r* ^2^ = 0.67, *p* = 0.09, dFn = 1, dFd = 3, *F* = 6.02; Chrimson = 0.16 ± 0.07, *r* ^2^ = 0.91, *p* = 0.09, dFn = 1, dFd = 3, *F* = 29.2; Chronos = 0.036 ± 0.007, *r* ^2^ = 0.66, *p* = 0.01, dFn = 1, dFd = 3, *F* = 5.91; Fig. 4*D–F*).

### Opsin-specific recruitment of cortical γ rhythms

**Figure 5.**
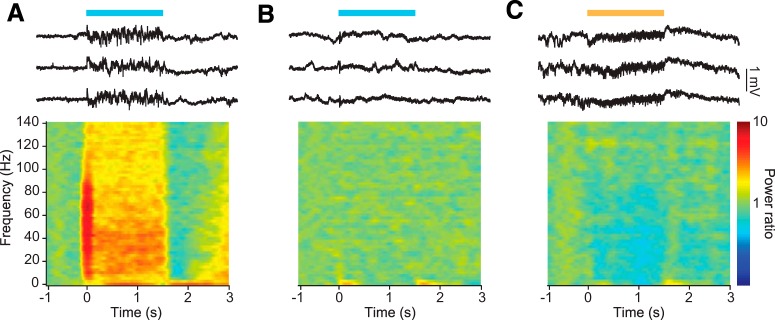
Different ChRs evoke distinct cortical activity profiles *in vivo*. ***A***, Example traces of cortical LFP activity in response to 1.5 s of 10 mW/mm^2^ light stimulation of ChR2-expressing pyramidal neurons (upper). Average changes in spectral power density at this site across stimulation trials (lower). ***B***, Same as in ***A***, for Chronos. ***C***, Same as in ***A***, for Chrimson.

Previous work has found that ChR2 stimulation of pyramidal neurons engages the cortical γ rhythm (30–80 Hz), an outcome of resonant excitatory-inhibitory circuit interactions (Cardin, 2016), *in vitro* and *in vivo* (Adesnik and Scanziani, 2010). Using evoked γ power as a measure of network activation, we assayed the efficacy of each optogenetic tool in driving recurrent circuit interactions. Activation of ChR2-expressing excitatory neurons evoked a response in the LFP and a broadband increase in high-frequency activity centered around the γ band (Fig. 5*A*). Chronos and Chrimson likewise evoked an initial deflection of the LFP signal (Fig. 5*B*,*C*). However, neither Chronos nor Chrimson activation of excitatory neurons evoked the characteristic sustained high-frequency LFP activity observed following ChR2 stimulation of the same population of neurons.

**Figure 6.**
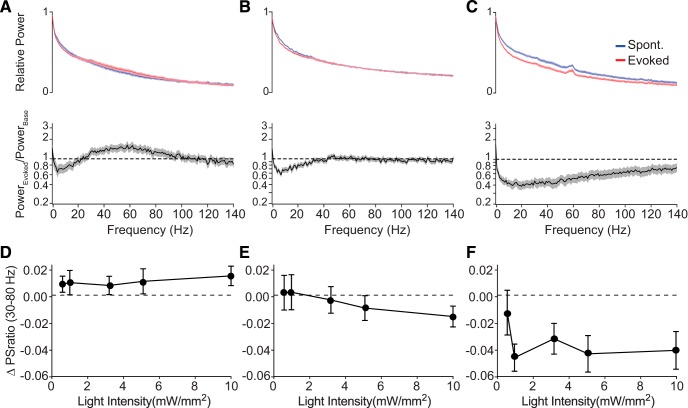
Distinct recruitment of γ-band activity by different ChRs. ***A***, Normalized power spectra (upper) for spontaneous (blue) and evoked (red) cortical LFP activity in response to ChR2 stimulation, averaged across all stimulation levels, and the ratio between evoked and spontaneous spectra (lower). ***B***, Same as in ***A***, for Chronos. ***C***, Same as in ***A***, for Chrimson. Shaded areas denote ± SEM. ***D***, Change in the relative power in the γ band (30–80 Hz) in response to varying light intensities in cortex expressing ChR2. ***E***, Same as in ***D***, for Chronos. ***F***, Same as in ***D***, for Chrimson. Error bars denote SEM.

In agreement with previous work (Adesnik and Scanziani, 2010), we found that the light-activation of ChR2-expressing excitatory neurons amplified LFP power in the γ range (*p* = 0.03, *W* = 19; Wilcoxon signed-rank test; Fig. 6*A*,*D*). In contrast, neither Chrimson nor Chronos demonstrated similar engagement of endogenous patterns of cortical network activity during stimulation. Stimulation of excitatory neurons via activation of Chronos had little effect on γ power (*p* = 0.23, *W* = –8; Fig. 6*B*,*E*). Surprisingly, excitatory neuron stimulation via Chrimson significantly suppressed cortical power across a range of high frequencies, including the γ band, in a light-intensity-dependent manner (*p* = 0.023, *W* = –15; Figs. 5*C*, 6*C*,*F*). Together, these data suggest that the pattern of activity recruited by stimulation with these three different tools engages distinct modes of endogenous circuit interactions.

## Discussion

Although recent work has resulted in the development of new ChR variants to meet experimental needs, the *in vivo* effects of opsins with distinct properties have not been fully explored. In particular, varying temporal profiles of optogenetically evoked neural activation may substantially affect the manner in which the surrounding neural circuit is engaged. Here, we expressed three opsins (ChR2, Chronos, and Chrimson) with different kinetics in excitatory pyramidal neurons in the primary visual cortex. Using a previously validated paradigm for optogenetic recruitment of γ-range resonance in the local cortical circuit, we compared the temporal envelope of evoked spiking and γ-range activity across the three opsins. Although all three tools were effective in driving enhanced spiking, the temporal profile of the evoked activity was distinct. In addition, only ChR2 stimulation generated increased cortical γ activity.

Recently developed ChRs vary extensively in their kinetic profiles. ChR2 exhibits a relatively fast onset (τ_on_) but a long offset time (τ_off_), leading to diminished temporal fidelity in spike responses (Gunaydin et al., 2010). The τ_off_ of several ChRs also slows further on membrane depolarization (Mattis et al., 2011). The cumulative effect of this long τ _off_ is to cause a prolonged depolarization after the evoked action potential, preventing rapid re-hyperpolarization of the membrane and contributing to artificial spike doublets. Prolonged depolarization may also inactivate voltage-gated channels needed for high-frequency spiking. In comparison, Chronos exhibits large photocurrents, rapid deactivation, and improved efficacy in eliciting high-fidelity fast spiking (Lin et al., 2009; Klapoetke et al., 2014).

Another recent series of tools were developed with absorption spectra shifted toward longer wavelengths compatible with two-photon imaging (Packer et al., 2012; Prakash et al., 2012; Agetsuma et al., 2017) and dual-channel optogenetic circuit interrogation. Chrimson exhibits a red-shifted absorption peak and very large photocurrents, making it highly effective for driving robust neural activity (Klapoetke et al., 2014). Chrimson has substantially slower τ_on_ and τ_off_ properties than ChR2, Chronos, ReaChR, or bReaCHES (Yizhar et al., 2011; Lin et al., 2013; Klapoetke et al., 2014; Rajasethupathy et al., 2015). Based on their low toxicity, robust expression levels, large peak photocurrents, and distinct kinetic profiles, we selected Chronos and Chrimson for *in vivo* comparison with ChR2.

We found a strong relationship between the properties of the individual opsins and the temporal profile of the spiking they evoked. The two tools with relatively rapid onset kinetics, ChR2 and Chronos, each evoked a precisely timed initial spike event across the neuronal population, followed by sustained spiking at lower firing rates. In contrast, Chrimson, with slow onset kinetics, did not evoke reliable spiking at stimulation onset and gave rise to a much broader temporal distribution of spike frequencies. These results, along with previous findings (Mattis et al., 2011; Schoenenberger et al., 2011), suggest that the kinetics of the opsins interact meaningfully with intrinsic neuronal membrane properties to affect the temporal pattern of evoked spiking. Rapid membrane depolarization, like that caused by ChR2 or Chronos activation, contributes to recruitment of voltage-gated channels and enhances the reliability and precision of the initial evoked action potentials in cortical neurons (Wilent and Contreras, 2005; Cardin et al., 2010). In comparison, a slow rate of depolarization, like that caused by Chrimson, leads to temporally dispersed spiking. We further found that the kinetics of the opsins shaped the overall temporal envelope of the sustained spiking evoked by long stimulation. Whereas the initial efficacy of ChR2 and Chronos resulted in an early peak in evoked firing rates within the first 50 ms, the spike response to Chrimson stimulation peaked several hundred milliseconds later. However, the sustained firing rates evoked by ChR2 and Chrimson were higher than that evoked by Chronos, suggesting that rapid deactivation of this opsin may reduce overall spike rates.

Gamma oscillations are generated by reciprocal, rhythmic interactions between excitatory and inhibitory neurons (Cardin, 2016). Gamma activity in cortical circuits can be evoked by optogenetically stimulating either the inhibitory interneurons (Cardin et al., 2009; Sohal et al., 2009; Veit et al., 2017) or the excitatory neurons (Adesnik and Scanziani, 2010; Lu et al., 2015). Generation of γ oscillations by excitatory neuron stimulation likely results from the highly synchronous activation of a large volley of spikes from excitatory neurons, which are particularly effective in activating the inhibitory neuron spiking that sets the temporal pattern for resonance in the network (Cardin et al., 2009; Sohal et al., 2009; Buzsáki and Wang, 2012; Cardin, 2016). Several cycles of γ can be produced by even a single brief stimulation of excitatory pyramidal neurons (Sohal et al., 2009), but sustained γ oscillations in active cortical networks *in vivo* may require consistently elevated excitatory spiking (Brunel and Wang, 2003; Buzsáki and Wang, 2012). Given these temporal constraints, the different temporal profiles of evoked excitatory neuron spiking evoked by the three ChRs could potentially engage varying network responses.

In good agreement with previous work (Adesnik and Scanziani, 2010), we found that ChR2 stimulation of excitatory pyramidal neurons evokes robust cortical γ activity. In contrast, stimulation of the same neuronal population via Chronos evoked little to no γ activity, presumably because the initial, highly precise spiking from stimulated cells is not followed by sufficiently elevated excitatory spiking to sustain network oscillations. In comparison, Chrimson might be expected to evoke little γ because the slow increase in activity precludes an initial burst of spikes. Surprisingly, we found that stimulation via Chrimson also significantly suppressed endogenous power in a broad range of high frequencies, including the γ band. These results suggest that Chrimson’s slow temporal kinetics and late firing peak destabilize the highly precise interplay between E and I cells, increasing the firing rates of excitatory neurons but broadly disrupting high-frequency activity and precluding rhythmic entrainment by inhibition. Because the impact of optogenetic drive to interneurons on oscillatory activity may vary with behavioral state (Veit et al., 2017), further work may be necessary to determine whether these findings, obtained under a low level of anesthesia, extend to other states such as wakefulness.

Overall, we found that differences in the properties of three ChRs were associated with distinct profiles of evoked cortical activity. Although this does not represent an exhaustive evaluation of all available opsins, our data suggest that the temporal properties of the opsins affect the temporal profile of evoked activity on multiple time scales. Rapid onset kinetics may facilitate the recruitment of highly precise initial spike responses, whereas slow onset kinetics preclude synchronous spiking and result in delayed peak responses. In addition, opsins with distinct kinetics interact differently with endogenous circuit resonance, affecting the sustained patterns of activity evoked in cortical networks over longer time periods. Our findings suggest complex interactions between optogenetic tools and active neuronal networks in the intact brain. The optogenetics toolkit for neuroscience includes an ever-increasing variety of tools with varying properties, and individual tools may be appropriate for different experimental goals.

## Synthesis

Reviewing Editor: Lorna Role, NINDS Decisions are customarily a result of the Reviewing Editor and the peer reviewers coming together and discussing their recommendations until a consensus is reached. When revisions are invited, a fact-based synthesis statement explaining their decision and outlining what is needed to prepare a revision will be listed below. The following reviewer(s) agreed to reveal their identity: Thomas Knopfel.

The reviewers felt that this revised version of the report was significantly improved, especially with respect to the statistics. The results of this study will be of great use to the number of labs using different versions of opsins to activate neurons and casts doubt on the canonical nature of “activation.”

Nevertheless there are still some concerns pertaining to issues that were partially but not completely addressed. We ask that these “Major issues” are addressed for resubmission.

Overall, the reviewers felt that the study merits the additional work requested so that the importance of the cautionary note that the report provides will be fully appreciated. Specifically ( to quote one of the reviewers directly, which I seldom do) “I believe that the issue they raise is of extreme importance to the Neuroscience field. If they provide [less than compelling] evidence, the paper will be discarded, and the field will not engage into corrective action. If the message is strong, it will have an impact.”

MAJOR ISSUES

1. Statistical Analyses:

While the statistical tests are more appropriate, the use of estimation statistics is needed and determination of effect size would be very useful. (please see Commentary by Jageman, in eNeuro on the use and importance of estimation stats) In addition to p values, please include F-, H-, etc. statistics and degrees of freedom for all tests.

2. Interpretation of Time Frequency data

In Fig. 5, there are obvious differences in the time-frequency plots for the three variants.

The plots reported in Fig. 6 show an enhancement in gamma for a, no change for b, and a general broadband suppression in c. The authors continue to interpret c as a suppression of gamma, when it is not. There is no identifiable gamma power in the original spectrum for c and the suppression is for all frequencies, including those above and below the gamma band. Yes, there is lower power in what is called the gamma band, but when neither spectrum shows any deviation from the 1/f<alpha> falloff of power (plotted on a log-log plot, it would be a straight line), there is no evidence of gamma. To report this as gamma suppression is misinterpretation of the data. Perhaps the example is a bad one, and there are other examples in which the original spectrum in c has an obvious increase in power specific to the gamma band. If this is the case, please show it or else reinterpret this effect as a general suppression of broadband power.

3. Analysis of effects of varying intensity.

The same intensity may yield different effects as the actual delivery of light will be different for different wavelengths. I/O curves would be best to analyze the effects completely. Also, the effects may be brain-state specific, as brain states fluctuate between different microstates, with different properties. If the authors do not have a full I/O analysis, perhaps the issue of different intensities could be added to the discussion

ADDITIONAL TOPICS THAT CAME OUT OF THE SYNTHESIS DISCUSSIONS

The reviewers felt it might be relevant to include a discussion of how different approaches to the analysis of the time frequency data and the placement of the reference may affect the interpretation of these types of data. Specifically, the points that were raised include:

a. Reference placement:

The reviewers discussed the best choice and effects of reference placement as potentially relevant to results. One reviewer felt that the choice of cortical reference might be problematic, the other defended the choice as S.O.P. Several relevant papers came up in the back and forth toward the synthesis that you might consider including A paper from D. Robbe in eNeuro and a review by O. Herreras in Frontiers.

b. Spectrum analysis:

There was a discussion about how using fft with a sliding window can provide false positives and negatives and a suggestion that one should whiten the signal and perform a wavelet decomposition. On the other hand, it was pointed out that a even direct comparisons of results from wavelet vs half overlapping standard tapered, and multi-taper analysis has given slightly different answers but still in a similar range. It was concluded that although wavelets is not the perfect technique, it may be less subject to artifact when the signal varies in time.

As you can see your paper generated an interesting and dynamic discussion. Perhaps you can incorporate these additional ideas into your discussion. At the very least I hope that they are helpful to you in thinking about the data.

## Author Response

We thank the reviewers for their effort to improve the manuscript. We are very encouraged by their level of enthusiasm, and we feel the study has been greatly clarified and improved.

---------------------------------------------

Synthesis of Reviews:

Computational Neuroscience Model Code Accessibility Comments for Author (Required):

n/a

Significance Statement Comments for Author (Required):

n/a

Comments on the Visual Abstract for Author (Required):

n/a

Synthesis Statement for Author (Required):

The reviewers felt that this revised version of the report was significantly improved, especially with respect to the statistics. The results of this study will be of great use to the number of labs using different versions of opsins to activate neurons and casts doubt on the canonical nature of “activation.”

Nevertheless there are still some concerns pertaining to issues that were partially but not completely addressed. We ask that these “Major issues” are addressed for resubmission.

Overall, the reviewers felt that the study merits the additional work requested so that the importance of the cautionary note that the report provides will be fully appreciated. Specifically ( to quote one of the reviewers directly, which I seldom do) “I believe that the issue they raise is of extreme importance to the Neuroscience field. If they provide [less than compelling] evidence, the paper will be discarded, and the field will not engage into corrective action. If the message is strong, it will have an impact.”

MAJOR ISSUES

1. Statistical Analyses:

While the statistical tests are more appropriate, the use of estimation statistics is needed and determination of effect size would be very useful. (please see Commentary by Jageman, in eNeuro on the use and importance of estimation stats) In addition to p values, please include F-, H-, etc. statistics and degrees of freedom for all tests.

We have added estimation statistics to all the statistical tests listed in the manuscript.

2. Interpretation of Time Frequency data

In Fig. 5, there are obvious differences in the time-frequency plots for the three variants.

The plots reported in Fig. 6 show an enhancement in gamma for a, no change for b, and a general broadband suppression in c. The authors continue to interpret c as a suppression of gamma, when it is not. There is no identifiable gamma power in the original spectrum for c and the suppression is for all frequencies, including those above and below the gamma band. Yes, there is lower power in what is called the gamma band, but when neither spectrum shows any deviation from the 1/f falloff of power (plotted on a log-log plot, it would be a straight line), there is no evidence of gamma. To report this as gamma suppression is misinterpretation of the data. Perhaps the example is a bad one, and there are other examples in which the original spectrum in c has an obvious increase in power specific to the gamma band. If this is the case, please show it or else reinterpret this effect as a general suppression of broadband power.

We agree with the reviewers that the effects in Figures 5 and 6 highlight a difference between the effects of ChR2 and Chrimson. Although ChR2 evokes enhanced power in the gamma band, Chrimson activation evokes a broadband suppression of high-frequency activity that includes the gamma range. We have updated the text in the results and discussion sections to more clearly reflect these findings.

3. Analysis of effects of varying intensity.

The same intensity may yield different effects as the actual delivery of light will be different for different wavelengths. I/O curves would be best to analyze the effects completely. Also, the effects may be brain-state specific, as brain states fluctuate between different microstates, with different properties. If the authors do not have a full I/O analysis, perhaps the issue of different intensities could be added to the discussion

The reviewers are correct that the impact of optogenetic manipulations on network activity varied with brain state and also potentially with wavelength. A complete I/O analysis accounting for all brain states is beyond the scope of the current study. In the current data set we can provide evidence only for the anesthetized state. In these experiments, we held the anesthesia at a low dose to generate a relatively desynchronized state without substantial contribution from up and down states associated with the slow oscillation. We have added text to the discussion to more fully highlight these limitations and potential future experiments that might be relevant to these findings.

ADDITIONAL TOPICS THAT CAME OUT OF THE SYNTHESIS DISCUSSIONS

The reviewers felt it might be relevant to include a discussion of how different approaches to the analysis of the time frequency data and the placement of the reference may affect the interpretation of these types of data. Specifically, the points that were raised include:

a. Reference placement:

The reviewers discussed the best choice and effects of reference placement as potentially relevant to results. One reviewer felt that the choice of cortical reference might be problematic, the other defended the choice as S.O.P. Several relevant papers came up in the back and forth toward the synthesis that you might consider including A paper from D. Robbe in eNeuro and a review by O. Herreras in Frontiers.

In our experience, a local cortical reference is fairly standard for local field potential recordings. Reference location will have an impact on the detectability and amplitude of activity with different origins, such as oscillations that are generated by local versus long-range synaptic interactions. In the current study, we are examining an artificially generated activity pattern whose source is local due to the optogenetic activation of specific local neurons and whose circuit-level mechanisms have been previously characterized. We have added relevant citations to some excellent reviews on this topic in the methods, but we feel that an extensive discussion is outside the scope of the current paper.

b. Spectrum analysis:

There was a discussion about how using fft with a sliding window can provide false positives and negatives and a suggestion that one should whiten the signal and perform a wavelet decomposition. On the other hand, it was pointed out that a even direct comparisons of results from wavelet vs half overlapping standard tapered, and multi-taper analysis has given slightly different answers but still in a similar range. It was concluded that although wavelets is not the perfect technique, it may be less subject to artifact when the signal varies in time.

We agree with the reviewers that a sliding fft can be prone to introducing errors under some circumstances. However, we note that the STFT analysis was used only in generating the visualizations in Figure 5, and not in the quantitative analysis of LFP signals.

As you can see your paper generated an interesting and dynamic discussion. Perhaps you can incorporate these additional ideas into your discussion. At the very least I hope that they are helpful to you in thinking about the data.

We were very encouraged by the suggestions of the reviewers at each stage, and we appreciate their efforts to improve the manuscript.
